# Some Haematological and Biochemical Investigations on Duck Virus Hepatitis following Administration of Glycyrrhizin

**DOI:** 10.1155/2013/849412

**Published:** 2013-07-30

**Authors:** Faten A. Okda, Safaa Yassein, Alaa R. Ahmed, Hamdy Soufy, Soad M. Nasr

**Affiliations:** ^1^Department of Parasitology and Animal Diseases, National Research Center, El-Behouse Street, Dokki, P.O. Box 12622, Giza, Egypt; ^2^Department of Clinical Pathology, Faculty of Veterinary Medicine, Cairo University, P.O. Box 12211, Giza, Egypt

## Abstract

The present study aimed to investigate the protective effect of glycyrrhizin (locally isolated and purified from licorice root) against duck hepatitis virus through the assessment of some hematological and biochemical parameters. One hundred and sixty white Pekin ducklings—one day old—were randomly divided into four equal groups. Group (1) was kept as normal control. Group (2) was inoculated I/P with 10 mg glycyrrhizin/kg BW, three times per week for four weeks. Group (3) was inoculated I/M with 0.5 ml of live attenuated DHV vaccine. Group (4) was inoculated with both glycyrrhizin (10 mg/kg BW I/P, three times per week for four weeks) and live attenuated DHV vaccine (0.5 ml, I/M). Then, all groups of treatment were challenged using virulent DHV except for 20 ducklings from the normal control group which were continued to be kept as negative control. The results revealed that duck hepatitis virus (DHV) caused macrocytic hypochromic anemia, leukopenia, hypoproteinemia, hypoalbuminemia, hyperglycemia, hypercholesterolemia, and marked elevation of liver enzymes and renal parameters. In conclusion, glycyrrhizin injected alone or in combination with DHV vaccine protected or ameliorated the deteriorating effects induced by DHV vaccine and/or duck hepatitis virus infection by improvement of erythrogram and leukogram, as well as liver and kidney functions.

## 1. Introduction

Duck virus hepatitis (DVH) is one of the most economic import diseases to all duck-growing farms because of its high potential mortality if the infection is not controlled. It is an acute highly fatal rapidly spreading viral infection of young ducklings. It was first recoded in New York and Taiwan. The morbidity is 100% and the mortality may reach 95–100%, in the first week of age [1].

Survived ducklings after DHV infection have a solid immunity, but it is necessary to protect the duck industry against such fatal disease using the potent-specific vaccine. Live attenuated DVH-1 vaccine which could be administrated through the intramuscular route in breeder ducks 2-3 weeks before lying allowing the transmission of high maternal immunity to the offspring providing them with passive immunity that is able to protect the new hatched birds up to 15 days of age. Also, it could be injected in 2-day-old ducklings followed by a booster dose 2-3weeks later [2].

In Egypt, duck farms are routinely protected against DVH following a vaccination program employing DVH-1 which is the only vaccine recorded in Egypt. Unfortunately, some duck farms stopped vaccination against DVH causing the recurrence of disease outbreaks [1].

Glycyrrhizin (GL) is the major active component extracted from licorice (*Glycyrrhiza glabra*) roots. GL has anti-inflammatory and antiviral effects that have been used in the treatment of patients with chronic hepatitis B and C [3–7]. GL enhances the production of antibodies through the production of interleukin- 1 (IL-1), IL-2, and IL-12 [8]. Soufy et al. [9] recorded that GL possesses a good immunostimulant and synergistic effect to DVH vaccine through activation of T lymphocyte proliferation. GL with vaccine enhances higher antibody titer against DHV than vaccine alone and high immunity protection.

The present study was conducted to investigate the protective effect of glycyrrhizin injected alone or in combination with live attenuated DHV vaccine against experimental infection of ducklings with virulent duck hepatitis virus through the assessment of some haematological and biochemical parameters.

## 2. Materials and Methods

The experiment was done according to guidelines for animal experimentations and approved by the Institutional Animal Care and Use Committee, National Research Center, Animal Care Unite, Dokki, Giza, Egypt.

### 2.1. Extraction, Purification, and Identification of Glycyrrhizin from Licorice Plant

Two kg of licorice root (*Glycyrrhiza glabra *L.) was obtained from Haraz, Abdeen, Cairo, Egypt, for extraction of glycyrrhizin (GL). Purification of glycyrrhizin from licorice was performed according to Bentley and Trimen [10]. The extracted and purified substances were identified using thin layer chromatography (TLC) according to the method of Cui et al. [11].

Glycyrrhizin was administrated as stronger neo-minophagen C (SNMC). It consists of purified glycyrrhizin 2% + cysteine 0.2% + glycine 2% dissolved in physiological saline. It was inoculated 3 times weekly for 4 weeks [12]. Cysteine and glycine were added to avoid side effect of glycyrrhizin by increasing glutathione synthesis and prevent the sodium and water retention effect.

### 2.2. Ducklings Used

One hundred and sixty white Pekin domestic ducklings one day old, obtained from a private nonvaccinated parent flock without maternal immunity were used in this study. Ducklings were housed under hygienic measures in separate isolators receiving a balanced growing broiler ration, containing protein 21%, fats 3.6%, and fibers 3.4% according to NRC [13].

### 2.3. Experimental Design

Ducklings were kept for 4 days for acclimatization and were allocated into 4 equal groups. Group (1) was kept as normal control (without glycyrrhizin treatment or vaccination). Group (2) was inoculated intraperitoneally (I/P) with 10 mg of glycyrrhizin/kg BW as SNMC (3 times weekly for 4 weeks). Group (3) was inoculated intramuscularly (I/M) with 0.5 mL of live attenuated DHV vaccine (obtained kindly from Vet Serum and Vaccine Research Institute, Abbassia, Cairo). Group (4) was inoculated with glycyrrhizin as SNMC (10 mg/kg BW, I/P, 3 times weekly for 4 weeks) and live attenuated DHV vaccine (0.5 mL) I/M. At the end of the 3rd week, all groups were challenged except 20 ducklings from the normal control group were continued to be kept as negative control.

### 2.4. Hemogram and Biochemical Analysis

Blood samples were collected from each duckling by jugular vein puncture before the challenge test at the end of the 1st, 2nd, and 3rd weeks and after the challenge at the 4th week of the beginning of the experiment. Each blood sample was divided into two portions; the first one was anticoagulated by disodium salt of ethylene diamine tetra acetic acid (EDTA) for determination of hemogram [14]. The second portion was placed in a plain centrifuge tube for serum separation and determination of biochemical constituents; total proteins [15], albumin [16], activities of aspartate aminotransferase (AST), alanine aminotransferase (ALT) [17], alkaline phosphatase (ALP) [18], glucose [19], creatinine [20], uric acid [21], and cholesterol [22]. Test kits supplied by bioMérieux-France were used.

### 2.5. Challenge Test

The ducklings of each group at the end of the 3rd week of treatment (at the 25th day of age) were challenged I/M with 0.5 mL virulent DHV containing 10^7^ TCID_50_ per duckling. The strain was kindly supplied from Vet Serum and Vaccine Research Institute, Abassia, Cairo.

### 2.6. Statistical Analysis

Results are expressed as the mean ± SD. Differences between control and treated groups and differences between control-infected group and other groups after challenge were tested for significance using a one-way analysis of variance followed by least significant difference (LSD). Differences were considered significant at *P* < 0.05 level [23] using SPSS version 10 computer program.

## 3. Results

### 3.1. Clinical Signs

Before the challenge, ducklings of normal control and glycyrrhizin-treated groups (1 and 2) appeared healthy during the experimental period. However, vaccinated group (3) showed signs of illness in the form of depression, decreased food intake, ruffled feather, and dullness at the 2nd and 3rd days. These signs started to disappear at the 4th and 5th days after vaccination. On the other hand, ducklings of treated and vaccinated group (4) were slightly depressed, mildly anorexic at the 2nd and 3rd days after vaccination, and returned to normal at the 4th and 5th days.

After the challenge test (at the 25th day of age), control infected group of ducklings showed severe depression, ruffled feather, and off food. Some ducklings were lying on their sides or breast with leg extended backward and head drown over the back with spasmodic paddling leg movement on the 3rd, 4th, 5th, and 6th days after challenge. The morbidity rate reached 80%, and the mortality rate reached 70%. Glycyrrhizin group (2) showed slight depression and general signs of illness at the 2nd day after challenge; one duckling had spasmodic paddling leg movement but returned healthy at the 3rd day. Neither mortality nor morbidity was recorded, and ducklings appeared healthy with normal size. In addition, ducklings of vaccinated group (3) showed moderate depression and general signs of illness at the 2nd day after challenge; one duckling died at the 3rd day after challenge. The survived duckling was emaciated and maintained stunted growth. In contrast, high protection rate (100%), healthy appearance, normal growth, and size were observed in glycyrrhizin-treated and -vaccinated ducklings (group 4) as shown in Table 1.

### 3.2. Erythrogram

Compared to normal control group (1) before challenge, red blood cell count (RBC), packed cell volume (PCV) %, and haemoglobin (Hb) concentration showed significant increase in glycyrrhizin-treated group (2) from the 1st till the 3rd week. Glycyrrhizin-treated and vaccinated group (4) showed significant increases in RBCs count and PCV % at the 2nd week. Vaccinated group (3) showed significant decreases in the RBCs count, PCV %, and Hb concentration at the 1st and 2nd weeks. At the 4th week after challenge, RBCs count, PCV %, and Hb concentration showed significant decrease in all groups compared to the normal control group (Table 2).

Vaccinated group (3) showed significant an increase in mean corpuscular volume (MCV) and a significant decrease in mean corpuscular haemoglobin concentration (MCHC) at the 1st week before challenge in comparison with normal control group (1). At the 4th week after challenge, significant increase in MCV with marked decrease in MCHC was demonstrated in control-infected and vaccinated groups, while no significant changes were observed in glycyrrhizin-treated group (2) and glycyrrhizin-treated and -vaccinated group (4). This data revealed the presence of macrocytic hypochromic anemia at the 1st week in vaccinated group and at the 4th week (after challenge) in control-infected and vaccinated group (Table 2).

### 3.3. Leukogram

Before challenge, compared to normal control group (1), glycyrrhizin-treated group (2) showed significant leukocytosis all over the experimental period with lymphocytosis started from the 1st week and monocytosis started from the 2nd week till the end of the experiment. Vaccinated group (3) showed significant leukopenia at the 1st week associated with significant lymphocytosis at the 3rd week and monocytosis from the 1st week till the end of experiment, while significant decreases in heterophil and eosinophil count were detected from the 1st week till the end of the experiment. Glycyrrhizin-treated and -vaccinated group (4) showed leukocytosis, lymphocytosis, and monocytosis from 1st week till the end of the experiment (Table 3). At the 4th week (after challenge), compared to control negative group, significant leukopenia, heteropenia, and eosinopenia were detected in control-infected, vaccinated, treated and vaccinated, and treated groups, respectively, with significant lymphocytosis and monocytosis in glycyrrhizin-treated and -vaccinated, control-infected, and treated groups, respectively (Table 3).

### 3.4. Serum Proteins Profile

Before challenge, compared to normal control group (1), glycyrrhizin-treated group (2) showed significant increases in total proteins and globulins at the 2nd week with significant increases in albumin at the 2nd and the 3rd weeks. Vaccinated group (3) showed significant increase in total proteins with significant hyperglobulinemia at the 2nd week, while significant hypoalbuminemia was observed at the 1st and 2nd weeks with significant decrease in A/G ratio from 1st week till the end of the experiment. Glycyrrhizin-treated and -vaccinated group (4) showed significant increase in total proteins with significant hyperglobulinemia at the 2nd and 3rd weeks, while significant decreases were observed in A/G ratio from the 1st week till the end of the experiment except for significant increase at the 3rd week with no significant changes in albumin concentration (Table 4). At the 4th week (after challenge), control-infected group showed significant hypoproteinemia, hypoalbuminemia, and significant decreases in A/G ratio compared to normal control group (1). Glycyrrhizin-treated group (2) showed significant increase in total proteins with hyperglobulinemia and significant decrease in A/G ratio at the 4th week and no significant changes in albumin. Vaccinated group (3) and treated and vaccinated group (4) showed significant hyperglobulinemia, hypoalbuminemia, and significant decrease in A/G ratio at the 4th week (Table 4).

### 3.5. Serum Total Cholesterol

Before challenge, compared to normal control group (1) serum total cholesterol showed significant increase in vaccinated group (3) at the 1st week and significant decreases in glycyrrhizin-treated group (2) at the 3rd week, while no significant changes were noticed in glycyrrhizin-treated and -vaccinated group (4) (Table 4). At the 4th week (after challenge), compared to normal control group (1), control-infected group and vaccinated group (3) showed significant increase, and treated and vaccinated group (4) showed marked decrease, while treated group (2) showed no significant change in serum total cholesterol (Table 4).

### 3.6. Serum Glucose

Before challenge, compared to normal control group (1), there is a significant hyperglycemia in vaccinated and treated and vaccinated groups at the 1st week, while significant hypoglycemia was noticed in treated and treated and vaccinated groups at the 3rd week. At the 4th week (after challenge) compared to control group (1), there is a significant hyperglycemia in vaccinated, control-infected, treated and vaccinated, and treated groups, respectively (Table 4).

### 3.7. Serum Liver Enzymes

Before challenge, compared to normal control group (1), activities of AST and ALT showed significant increases in vaccinated groups (3) at the 1st and 2nd weeks. ALP activities showed significant increase in vaccinated group (3) from the 1st week till the 3rd week, and treated and vaccinated group (4) showed significant increase at the 1st week, while no significant change was observed in treated group (2). At the 4th week after challenge, compared to control group (1) there is a significant increase in AST, ALT, and ALP activities in control-infected group, vaccinated group (3), and treated and vaccinated group (4) as shown in Table 5.

### 3.8. Serum Creatinine and Uric Acid

Before challenge, compared to normal control group (1), vaccinated group (3) showed significant increase in uric acid and creatinine at the 1st week and at the 1st and 2nd weeks, respectively. On the other hand, no significant changes were noticed in treated group (2) and treated and vaccinated group (4).

After challenge, compared to control group (1), control-infected group and vaccinated group (3) showed significant increases in uric acid and creatinine at the 4th week. Treated and vaccinated group (4) showed significant increase in uric acid at the 4th week, while treated group (2) showed no significant changes (Table 5).

## 4. Discussion

The present work was adopted to investigate the effect of GL as an antiviral and immunostimulant against DHV. This study is based on the assessment of haematological and serum biochemical responses.

Glycyrrhizin (GL), the active principle of licorice (*Glycyrrhiza glabra*) has antiviral, anti-inflammatory, and immunostimulant effects [24].

In the present study, most of vaccinated group and treated & vaccinated group showed symptoms of depression, loss of appetite, ruffled feather, and dullness at the 2nd and 3rd days after vaccination and began to disappear at the 4th and 5th days after vaccination. This period gets along with the incubation period of the DVH which varied from 2 to 7 days and stress effect of vaccination. Similar results were reported by Asplin and Mclauchlan [25] and Mahdy [1]. The symptoms which were observed in treated & vaccinated group were less severe than those of vaccinated group may be referred to the immunostimulant and hepatoprotective effect by GL which might decrease the undesirable effect of live attenuated vaccine [26].

Regarding the results of the challenge test at the 25 day of age, the mortality rate was the highest in control-infected group as 70% of ducklings died at the 3rd, 4th, 5th, and 6th days after inoculation which could be attributed to the immunosuppressive and deteriorated effect of the virus on the liver, kidneys, and thymus gland. These results were parallel to these reported by Saif et al. [2] and Mahdy [1].

Vaccinated group showed 10% mortality which may be attributed to the protective effect of the vaccine, while treated group showed no mortality which may be due to the enterohepatic cycle of GL, antiviral, immunostimulant, anti-inflammatory, and antioxidant effects [24]. Treated and vaccinated group (4) showed no mortality or morbidity due to both efficiency and potancy of GL and vaccine in controlling the virus.

Before challenge, results of erythrogram showed significant decrease in vaccinated group (3) at the 1st week and macrocytic hypochromic anemia then normocytic normochromic anemia at the 2nd week. This result could be attributed to loss of RBCs due to extravasation in different organs particularly in the liver and spleen of ducklings as mentioned by Mahdy [1]. Erythrogram of treated and treated and vaccinated groups showed significant increase which could be attributed to the hepatostimulatory and hepatoprotective effect of GL leading to production of more RBCs by the bone marrow under control of erythropoietic factors released by hepatic cells [27].

After challenge, results of erythrogram showed varying types of anemia in all groups which may be attributed to hemorrhage effect of DHV and vaccine on liver and spleen as reported by Campbell and Coles [28] and Mahdy [1]. The presence of different types of anemia may be due to bone marrow response to these hemorrhages [14].

Results of leukogram revealed leukocytosis in treated group (2) and treated and vaccinated group (4) before challenge. These results are similar to those of Al-Okbi et al. [29], who reported that licorice extract increased the total leukocytic count in rat.

Leukopenia was demonstrated at the 1st week in vaccinated group (3) before challenge and in all groups after challenge which may be due to the depressing effect of DHV on leukopoietic parameters or the severe leukocytic infiltration in different organs. These results were in agreement with Latimer and Bienzle [30] and Mahdy [1] who reported that leukopenia may be due to some viral infections or live vaccine. Leukopenia was less significant in treated group (2) and treated and vaccinated group (4) which may be due to the immunostimulant effect of GL [9, 31]. The observed lymphocytosis with monocytosis in treated group (2) and treated and vaccinated group (4) may be due to the antigenic stimulation of GL through its stimulation to the mononuclear cells [32]. Moreover, lymphocytosis in ducklings may be secondary to antigenic stimulation by the virus [9], and also monocytosis may be observed in association with antigenic agent (infection) as it has phagocytic function [33].

Results of proteins profile revealed significant hyperproteinemia due to hyperglobulinemia at the 2nd week with significant hypoalbuminemia at the 1st and 2nd weeks in vaccinated group (3) before challenge. After challenge, significant hyperproteinemia due to hyperglobulinemia was observed at the 4th week which may be due to the effect of the virus or vaccine on the proliferation of B lymphocyte [33], while hypoalbuminemia may be due to the adverse effect of vaccine or virus on liver and kidneys. Treated group showed significant hyperproteinemia and hyperglobulinemia at the 2nd week before challenge. The hyperproteinemia may occur due to the effect of GL to preserve the normal functional status of liver [29, 34, 35]. After challenge, it showed significant hyperproteinemia with hyperglobulinemia which may be due to the immunopotentiating action of GL through activation of T cell and the effect of GL as an immune modulating and biological response modifier activities [31]. GL-treated and -vaccinated group showed significant hyperproteinemia at the 2nd week before challenge which may be attributed to the compensatory effect of GL on the deteriorating action of vaccine. After challenge, significant increased hypoalbuminemia was detected at the 4th week due to the effect of DHV infection. The hyperglobulinemia could be attributed to the immunopotentiating effect of GL and vaccine related to the proliferation of B lymphocyte which were converted to plasma cells producing immunoglobolins as a result of vaccine or viral infection [28, 33]. Furthermore, the control-infected group showed significant hypoproteinemia and hypoalbuminemia at the 4th week which may be attributed to the damaging effect of the virus on the liver and kidneys. The observed results in control-infected and vaccinated groups (3) get on the same hand with those obtained by Ahmed et al. [36], Ellakany et al. [37], and Mahdy [1].

Serum total cholesterol showed significant increase in vaccinated group (3) at the 1st and 4th weeks and in the control-infected group at the 4th week which may be attributed to the hepatic injury caused by the DHV or vaccine [38]. GL-treated group (2) showed significant decrease in total cholesterol at the 3rd week which may be due to the antioxidant effect of GL [24] or the effect of GL on increasing the lipid peroxides in liver [39]. Nonsignificant changes after challenge may be due to the hepatoprotective action and antiviral effect of GL [7, 40]. GL-treated and -vaccinated group (4) showed no significant changes in total cholesterol all over the experimental period except significant decrease at the 4th week which may be attributed to the damage of hepatocytes caused by DHV which bounds to lipids and proteins through subcellular fraction [41].

Before challenge, significant hyperglycemia was noticed in vaccinated and treated and vaccinated groups at the 1st week which may be attributed to the stress effect of vaccination which increases gluconeogenesis in the liver [42]. Treated and treated and vaccinated groups showed significant hypoglycemia at the 3rd week which may be due to the inhibitory action of licorice extract on the activity of 11 beta-hydroxysteroid dehydrogenase which converts cortisol to cortisone. This enzyme protects the mineralocorticoid in kidneys [43, 44]. After challenge, significant hyperglycemia was observed at the 4th week in all groups which may be attributed to the stress factor of infection which enhances the gluconeogenes and glycogenolysis in the liver [42]. These results disagree with Mahdy [1] who reported that DHV or vaccine causes hypoglycemia due to damage of liver which resulted in decreased hepatic gluconeogenesis [45].

Regarding liver enzymes, vaccinated group (3) showed significant increase in the activity of ALT and AST at the 1st and 2nd weeks with significant increase in the activity of ALP at the 1st, 2nd, and the 3rd weeks (before challenge) which may be due to the damaging effect of vaccine on liver as hepatic degeneration or necrosis causes leakage of these enzymes, so elevation of the serum levels of AST and ALT were noticed, while the obstruction in bile duct associated with bile duct hyperplasia causes elevation in ALP. After challenge, vaccinated group (3) and control infected groups showed significant increase in the activity of AST, ALT, and ALP at the 4th week which may be attributed to the damaging effect of DHV on liver hepatocyte and biliary canaliculi. This result was rather similar to that of Hochleithner [46], Ellakany et al. [37], and Mahdy [1].

Treated group (2) showed normal AST, ALP, and ALT patterns; which may be attributed to the antiviral, antioxidant, and hepatoprotective effects of GL as it enters the enterohepatic loop, excreted in bile then reabsorbed in the gut to recycle repeatedly through liver [26] or the action of GL in inhibiting the activation of phospholipase A2 [47].

Treated and vaccinated group (4) showed no significant changes in the activity of ALT and AST all over the experiment except a significant increase which was noticed at the 4th week. ALP showed significant increases at the 1st and 4th weeks. The increases in liver enzymes may be attributed to the damage effect of DHV on liver cells and canaliculi [1]. Results were less severe than that recorded in both vaccinated and control-infected groups. These results agreed with Al-Qarawi et al. [34] who reported hepatoprotective effect of GL in rat.

Vaccinated group showed significant increase in uric acid at the 1st and 4th weeks and in creatinine at the 1st, 2nd, and 4th weeks. Control-infected group showed significant increase in uric acid and creatinine at the 4th week. Treated and vaccinated group (4) showed no significant changes in uric acid and creatinine at the 1st week but significant increase in uric acid at the 4th week. The increase in uric acid may be due to the damaging effect of DHV or vaccine on kidneys [1, 37].

Finally, DHV infection caused some pathognomonic clinicopathological changes (macrocytic hypochromic anemia, leukopenia, hypoproteinemia, hypoalbuminemia, hyperglobulinemia, hyperglycemia, hypercholesterolemia, and marked elevation of liver enzymes and renal parameters. Live attenuated vaccine gives high protection against DHV but causes some deleterious effects on liver and kidneys. GL has the ability to reduce the adverse changes associated with vaccine or infection through reduction of severity of the clinical signs associated with duck hepatitis vaccine or infection, minimizing the mortalities by the virus, GL alone, or with vaccine can reduce the drastic elevation of liver enzymes with considerable improvement in erythrogram, protein pattern, total cholesterol and glucose, and duckling general health condition. GL gives high protection and prevents the deteriorating action of vaccine and DHV infection.

## 5. Conclusion

Glycyrrhizin injected alone or in combination with DHV vaccine protected or ameliorated the deteriorating effects induced by DHV vaccine and/or duck hepatitis virus infection by improvement of erythrogram and leukogram, as well as liver and kidney functions.

## Figures and Tables

**Table 1:** Morbidity, mortality, and protection rates in different experimental groups of ducklings after challenge at the 25th day of age.

Groups	Morbidity rate	Mortality rate/days after infection														Mortality rate	Protection rate
		1	2	3	4	5	6	7	8	9	10	11	12	13	14		
Control	80%	—	—	2	3	1	1	—	—	—	—	—	—	—	—	70%	30%
Glycyrrhizin	10%	—	—	—	—	—	—	—	—	—	—	—	—	—	—	0%	100%
Vaccinated	10%	—	—	1	—	—	—	—	—	—	—	—	—	—	—	10%	90%
Glycyrrhizin and vaccinated	0%	—	—	—	—	—	—	—	—	—	—	—	—	—	—	0%	100%

**Table 2:** Erythrogram of different experimental groups of ducklings before (at the 1st, 2nd, and the 3rd weeks) and after challenge (at the 4th week) (mean ± standard deviation).

		Groups					
Parameters	Periods (weeks)	Control (1)		(2)	(3)	(4)	LSD
		Normal	After challenge	Glycyrrhizin	DHV vaccine	Glycyrrhizin + DHV vaccine	
Red blood cell count (10^6^/*µ*L)	1	2.64 ± 0.26		3.03* ± 0.19	1.95* ± 0.13	2.81 ± 0.33	0.32
	2	2.93 ± 0.39		3.30* ± 0.44	2.59* ± 0.26	3.23* ± 0.04	0.30
	3	3.01 ± 0.43		3.40* ± 0.29	2.87 ± 0.12	3.21 ± 0.14	0.37
	4^#^	3.04^b^ ± 0.40	1.75* ± 0.10	2.39^∗b^ ± 0.08	2.11^∗b^ ± 0.11	2.30^∗b^ ± 0.26	0.33

Packed cell volume (%)	1	37.32 ± 1.84		43.08* ± 2.55	33.40* ± 2.96	39.20 ± 1.92	3.12
	2	38.20 ± 1.78		44.84* ± 1.88	35.10* ± 2.1	40.84* ± 1.88	2.60
	3	39.00 ± 1.58		44.60* ± 3.84	37.80 ± 1.92	41.40 ± 1.94	3.30
	4^#^	39.20^b^ ± 1.48	29.00* ± 1.05	33.72^∗b^ ± 2.48	33.00^∗b^ ± 0.70	34.00^∗b^ ± 2.54	2.62

Hemoglobin (g/dL)	1	12.64 ± 0.21		14.68* ± 0.20	10.51* ± 0.35	13.08 ± 0.63	0.52
	2	13.04 ± 0.15		15.09* ± 0.09	10.76* ± 0.43	13.33 ± 0.75	0.59
	3	13.13 ± 0.25		15.00* ± 0.35	12.56 ± 0.65	13.79 ± 0.18	0.68
	4^#^	13.14^b^ ± 0.62	7.50* ± 1.25	10.58^∗b^ ± 0.94	9.28^∗b^ ± 0.64	10.48^∗b^ ± 0.45	0.91

Mean corpuscular volume (fL)	1	151.32 ± 14.02		142.49 ± 3.69	171.00* ± 11.15	142.50 ± 3.63	12.50
	2	132.84 ± 5.07		135.39 ± 8.69	132.80 ± 11.81	133.75 ± 10.26	12.47
	3	130.77 ± 5.58		130.10 ± 8.61	129.94 ± 10.96	128.88 ± 5.44	10.70
	4^#^	140.33^b^ ± 8.11	165.71* ± 8.11	142.21^b^ ± 9.22	162.20* ± 15.46	144.54^b^ ± 9.22	14.47

MCHC (g/dL)	1	33.51 ± 1.89		34.16 ± 2.99	30.65* ± 1.33	32.52 ± 1.31	2.81
	2	34.29 ± 0.44		34.17 ± 2.79	32.63 ± 1.73	3.26 ± 0.77	2.15
	3	33.78 ± 0.50		33.34 ± 1.07	33.22 ± 0.67	33.26 ± 0.77	1.05
	4^#^	33.17^b^ ± 1.25	25.86* ± 1.20	31.52^b^ ± 3.21	27.82* ± 1.02	30.96^b^ ± 1.07	2.51

4^#^ After challenge (at the 4th week).

*Significant compared to control normal group in the same row.

^b^Significant compared to control-infected group at the 4th week.

DHV: duck hepatitis virus.

LSD: least significant difference.

MCHC: mean corpuscular hemoglobin concentration.

**Table 3:** Leukogram of different experimental groups of ducklings before (at the 1st, 2nd, and the 3rd weeks) and after challenge (at the 4th week) (mean ± standard deviation).

		Groups					
Parameters	Periods (weeks)	Control (1)		(2)	(3)	(4)	LSD
		Normal	After challenge	Glycyrrhizin	DHV vaccine	Glycyrrhizin + DHV vaccine	
Total leukocytic count (10^3^/*µ*L)	1	26.76 ± 0.26		36.80* ± 0.64	21.80* ± 0.30	34.00* ± 0.70	1.70
	2	30.56 ± 1.04		37.00* ± 0.44	29.24 ± 1.22	35.28* ± 0.41	1.60
	3	34.44 ± 1.08		40.12* ± 1.50	34.56 ± 1.44	37.20* ± 1.48	1.86
	4^#^	33.80^b^ ± 4.08	22.00* ± 0.1	29.80^∗b^ ± 0.64	22.80* ± 0.30	28.88^∗b^ ± 0.70	3.69

Lymphocyte count (×10^3^/*µ*L)	1	20.29 ± 2.5		27.24* ± 2.02	19.04 ± 4.55	28.06* ± 4.34	4.74
	2	24.72 ± 3.72		31.70* ± 2.70	25.93 ± 3.39	35.75* ± 6.18	5.64
	3	24.91 ± 4.16		36.61* ± 2.62	30.68* ± 2.27	36.92* ± 2.54	4.01
	4^#^	17.00^b^ ± 2.27	19.02* ± 1.00	25.24^∗b^ ± 1.02	25.80^∗b^ ± 4.55	24.06^∗b^ ± 4.34	1.89

Heterophil count (×10^3^/*µ*L)	1	4.34 ± 0.33		4.57 ± 0.19	0.00* ± 0.41	4.20 ± 1.88	1.32
	2	4.30 ± 0.62		2.00 ± 0.00	0.00* ± 0.1	2.46 ± 1.88	0.42
	3	8.04 ± 1.02		0.21 ± 0.00	0.35* ± 1.02	0.00 ± 0.51	0.78
	4^#^	10.70^b^ ± 0.72	0.00* ± 0.00	1.27^∗b^ ± 0.00	0.40* ± 0.70	2.00^∗b^ ± 0.00	0.49

Monocyte count (×10^3^/*µ*L)	1	0.94 ± 0.00		1.02 ± 0.00	2.76* ± 0.28	1.45* ± 0.02	0.19
	2	1.01 ± 0.25		3.09* ± 0.00	2.36* ± 0.15	1.93* ± 0.06	0.20
	3	1.47 ± 0.00		3.07* ± 0.00	2.45* ± 0.00	1.64* ± 0.00	0.10
	4^#^	1.00^b^ ± 0.00	2.81* ± 0.98	2.46^∗b^ ± 0.00	3.77^∗b^ ± 0.00	2.03^∗b^ ± 0.00	0.10

Eosinophil count (×10^3^/*µ*L)	1	1.13 ± 0.03		1.00 ± 0.00	0.00* ± 0.00	0.25 ± 0.15	0.10
	2	0.32 ± 0.18		0.21 ± 0.00	0.00* ± 0.00	0.00 ± 0.00	0.12
	3	0.52 ± 0.00		0.08 ± 0.10	0.38* ± 0.01	0.00 ± 0.00	0.07
	4^#^	6.20^b^ ± 0.00	0.21* ± 0.20	0.25^∗b^ ± 0.00	0.02^∗b^ ± 0.00	0.37^∗b^ ± 0.00	0.00

4^#^ After challenge (at the 4th week).

*Significant compared to control normal group in the same row.

^b^Significant compared to control-infected group at the 4th week.

DHV: duck hepatitis virus.

LSD: least significant difference.

**Table 4:** Serum protein profile, total cholesterol, and glucose of different experimental groups of ducklings before (at the 1st, 2nd, and 3rd weeks) and after challenge (at the 4th week) (mean ± standard deviation).

		Groups					
Parameters	Periods (weeks)	Control (1)		(2)	(3)	(4)	LSD
		Normal	After challenge	Glycyrrhizin	DHV vaccine	Glycyrrhizin + DHV vaccine	
Total proteins (g/dL)	1	4.41 ± 0.71		4.75 ± 0.23	4.39 ± 0.57	4.65 ± 0.08	0.63
	2	4.07 ± 0.21		4.77* ± 0.34	4.97* ± 0.02	4.87* ± 6.85	0.67
	3	4.65 ± 0.36		5.07 ± 0.04	4.46 ± 0.37	5.14* ± 0.05	0.61
	4^#^	4.26^b^ ± 0.04	3.57* ± 0.29	5.14^∗b^ ± 0.39	4.62 ± 0.78^b^	4.68^b^ ± 0.34	0.58

Albumin (g/dL)	1	1.83 ± 0.21		1.93 ± 0.18	1.54* ± 0.17	1.70 ± 0.00	0.23
	2	1.84 ± 0.12		2.19* ± 0.23	1.53* ± 0.02	1.84 ± 0.13	0.20
	3	1.68 ± 0.29		2.05* ± 0.21	1.55 ± 0.12	1.87 ± 0.14	0.27
	4^#^	2.04^b^ ± 0.05	1.39* ± 0.13	1.85^b^ ± 0.30	1.34* ± 0.08	1.72^∗b^ ± 0.43	0.20

Globulins (g/dL)	1	2.58 ± 0.45		2.82 ± 0.11	2.83 ± 0.43	2.94 ± 0.32	0.47
	2	2.22 ± 0.10		2.63* ± 0.00	3.43* ± 0.28	2.98* ± 0.01	0.20
	3	2.96 ± 0.36		3.01 ± 0.02	2.92 ± 0.37	3.22* ± 0.04	0.22
	4^#^	2.22 ± 0.07	2.42 ± 0.01	3.29^∗b^ ± 0.45	3.43^∗b^ ± 0.30	2.83^∗b^ ± 0.28	0.37

A/G ratio	1	0.71 ± 0.04		0.63 ± 0.10	0.55* ± 0.06	0.54* ± 0.05	0.10
	2	0.82 ± 0.03		0.83 ± 0.03	0.45* ± 0.04	0.55* ± 0.09	0.20
	3	0.62 ± 0.03		0.65 ± 0.02	0.53* ± 0.06	0.89* ± 0.01	0.04
	4^#^	0.92^b^ ± 0.53	0.45* ± 0.00	0.57^∗b^ ± 0.02	0.35* ± 0.03	0.80^∗b^ ± 0.04	0.10

Total cholesterol (mg/dL)	1	259.18 ± 11.64		252.62 ± 19.59	294.88* ± 42.32	265.55 ± 20.30	34.98
	2	289.15 ± 16.11		257.25 ± 23.89	255.61 ± 37.09	258.98 ± 21.26	34.56
	3	257.44 ± 21.51		226.12* ± 16.57	252.33 ± 15.03	254.08 ± 13.29	22.63
	4^#^	362.70 ± 89.39	435.95* ± 55.62	325.88^b^ ± 55.09	442.07* ± 36.01	281.35^∗b^ ± 58.20	81.00

Glucose (mg/dL)	1	226.88 ± 27.62		237.00 ± 45.71	292.23* ± 14.08	278.29* ± 32.67	43.02
	2	236.25 ± 19.99		218.56 ± 1.96	234.50 ± 26.89	219.88 ± 18.97	25.85
	3	230.76 ± 12.69		166.47* ± 39.09	202.01 ± 14.47	190.35* ± 36.10	37.94
	4^#^	194.35^b^ ± 7.57	300.00* ± 9.97	266.88* ± 28.72	308.23* ± 59.65	296.35* ± 44.10	47.60

4^#^ After challenge (at the 4th week).

*Significant compared to control normal group in the same row.

^b^Significant compared to control-infected group at the 4th week.

DHV: duck hepatitis virus.

LSD: least significant difference.

**Table 5:** Serum enzymes, creatinine, and uric acid of different experimental groups of ducklings before (at the 1st, 2nd, and 3rd weeks) and after challenge (at the 4th week) (mean ± standard deviation).

		Groups					
Parameters	Periods (weeks)	Control (1)		(2)	(3)	(4)	LSD
		Normal	After challenge	Glycyrrhizin	DHV vaccine	Glycyrrhizin + DHV vaccine	
Aspartate aminotransferase (IU/L)	1	188.47 ± 7.36		177.00 ± 13.61	228.47* ± 1.08	194.00 ± 0.00	10.39
	2	186.86 ± 2.28		186.93 ± 3.58	195.48* ± 1.48	181.56 ± 9.80	7.23
	3	153.71 ± 5.88		140.56 ± 5.87	146.17 ± 17.92	142.20 ± 6.06	13.85
	4^#^	174.59^b^ ± 3.42	350.07* ± 13.33	179.95^b^ ± 10.72	261.20^∗b^ ± 17.78	228.13^∗b^ ± 2.84	14.82

Alanine aminotransferase (IU/L)	1	180.44 ± 27.03		189.10 ± 7.80	252.38* ± 27.77	186.36 ± 4.36	26.66
	2	184.65 ± 5.08		171.88 ± 0.06	198.11 ± 17.80	184.82 ± 1.28	12.44
	3	181.66 ± 18.23		149.73 ± 12.70	186.75 ± 4.85	176.37 ± 27.32	23.83
	4^#^	182.48^b^ ± 4.23	290.84* ± 27.35	192.84^b^ ± 9.83	235.60^∗b^ ± 14.42	210.52^∗b^ ± 12.88	20.78

Alkaline phosphate (IU/L)	1	166.97 ± 15.58		161.01 ± 10.18	264.98* ± 18.78	90.67* ± 14.55	0.24
	2	152.33 ± 3.11		147.80 ± 10.97	166.05* ± 9.08	127.93 ± 12.65	12.94
	3	153.35 ± 5.36		146.02 ± 4.78	162.94* ± 5.23	139.52 ± 8.33	8.16
	4^#^	156.39^b^ ± 3.93	251.59* ± 24.47	171.66^b^ ± 7.89	202.00^∗b^ ± 16.67	211.23^∗b^ ± 23.61	22.99

Creatinine (mg/dL)	1	1.68 ± 0.02		1.74 ± 0.04	2.24* ± 0.05	2.08 ± 0.01	0.47
	2	1.02 ± 0.02		1.22 ± 0.03	2.54* ± 0.03	1.33 ± 0.18	0.36
	3	1.41 ± 0.04		1.39 ± 0.04	1.83 ± 0.10	1.78 ± 0. 04	0.47
	4^#^	1.59^b^ ± 0.04	4.30* ± 0.64	1.94^b^ ± 0.32	3.55^∗b^ ± 0.27	2.08^∗b^ ± 0.39	0.59

Uric acid (mg/dL)	1	2.68 ± 0.48		4.23 ± 0.33	6.25* ± 1.87	3.99 ± 0.33	1.59
	2	4.78 ± 0.15		3.00 ± 0.13	4.01 ± 0.17	2.96 ± 0.09	2.04
	3	3.04 ± 0.40		2.61 ± 0.21	3.64 ± 0.10	3.95 ± 0.4	1.32
	4^#^	7.93^b^ ± 1.79	21.54* ± 1.54	6.01^**b**^ ± 1.55	21.26* ± 1.32	16.68* ± 3.17	9.76

4^#^ After challenge (at the 4th week).

*Significant compared to control normal group in the same row.

^b^Significant compared to control-infected group at the 4th week.

DHV: duck hepatitis virus.

LSD: least significant difference.

## References

[1] Mahdy SA (2005). *Clinicopathological studies on the effect of duck viral hepatitis in ducks [M.V.Sc. thesis]*.

[2] Saif YM, Barnes HJ, Glissons JR, Fadly AM, McDougald LR, Swayne DE (2003). *Diseases of Poultry*.

[3] Numazaki K, Nagata N, Sato T, Chiba S (1993). Effect of glycyrrhizin, cyclosporin A, and tumor necrosis factor *α* on infection of U-937 and MRC-5 cells by human cytomegalovirus. *Journal of Leukocyte Biology*.

[4] Fujisawa Y, Sakamoto M, Matsushita M, Fujita T, Nishioka K (2000). Glycyrrhizin inhibits the lytic pathway of complement—possible mechanism of its anti-inflammatory effect on liver cells in viral hepatitis. *Microbiology and Immunology*.

[5] Matsui S, Sonoda Y, Sekiya T, Aizu-Yokota E, Kasahara T (2006). Glycyrrhizin derivative inhibits eotaxin 1 production via STAT6 in human lung fibroblasts. *International Immunopharmacology*.

[6] Yoshida T, Abe K, Ikeda T (2007). Inhibitory effect of glycyrrhizin on lipopolysaccharide and d-galactosamine-induced mouse liver injury. *European Journal of Pharmacology*.

[7] Ogiku M, Kono H, Hara M, Tsuchiya M, Fujii H (2011). Glycyrrhizin prevents liver injury by inhibition of high-mobility group box 1 production by kupffer cells after ischemia-reperfusion in rats. *Journal of Pharmacology and Experimental Therapeutics*.

[8] Dai JH, Iwatani Y, Ishida T (2001). Glycyrrhizin enhances interleukin-12 production in peritoneal macrophages. *Immunology*.

[9] Soufy H, Yassein S, Ahmed AR (2012). Antiviral and immune stimulant activities of glycyrrhizin against duck hepatitis virus. *African Journal of Traditional, Complementary and Alternative Medicines*.

[10] Bentley R, Trimen H (1880). *Medicinal Plants. Extractum Glycyrrhiza (U.S.P). Extract of Glycyrrhiza*.

[11] Cui S, Fu B, Lee FS, Wang X (2005). Application of microemulsion thin layer chromatography for the fingerprinting of licorice. *Journal of Chromatography B*.

[12] Mori K, Sakai H, Suzuki S (1990). Effects of glycyrrhizin (SNMC: stronger neo-minophagen C) in hemophilia patients with HIV-1 infection. *Tohoku Journal of Experimental Medicine*.

[13] NRC (National Research Council) (1994). *Nutrient Requirements of Poultry*.

[14] Feldman BF, Joseph G, Zinkl JG, Jain NC (2000). *Schalm's Veterinary Hematology*.

[15] Henary RJ, Cannon DC, Winkleman JW (1974). *Clinical Chemistry Principles and Techniques*.

[16] Doumas BT, Watson WA, Biggs HG (1971). Albumin standards and the measurement of serum albumin with bromcresol green. *Clinica Chimica Acta*.

[17] Reitman S, Frankel S (1957). A colorimetric method for the determination of serum glutamic oxalacetic and glutamic pyruvic transaminases. *American Journal of Clinical Pathology*.

[18] Tietz NN (1976). *Fundamentals of Clinical Chemistry*.

[19] Trinder P (1969). Determination of serum glucose by enzymatic method. *Annals of Clinical Biochemistry*.

[20] Houot O (1985). *Interpretation of Clinical Laboratory Tests*.

[21] Trivedi RC, Rebar L, Berta E, Stong L (1979). New enzymatic method for serum uric acid at 500 nm. *Clinical Chemistry*.

[22] Watson D (1960). A simple method for the determination of serum cholesterol. *Clinica Chimica Acta*.

[23] Snedecor GW, Cochran WG (1982). *Statistical Methods*.

[24] Aviram M (2004). Flavonoids rich nutrients with potent antioxidant activity prevent atherosclerosis development, the licorice example. *International Congress Series*.

[25] Asplin FD, Mclauchlan JD (1954). Duck virus hepatitis. *Veterinary Record*.

[26] Murry M, Pizzorno J (1992). *A Textbook of Natural Medicine*.

[27] Sarma M, Sapcota D, Sarma S, Gohain AK (2003). Herbal growth promoters on haemato-biochemical constituents in broilers. *Indian Veterinary Journal*.

[28] Campbell TW, Coles EH, Coles EH (1986). Avian Clinical Pathology. *Veterinary Clinical Pathology*.

[29] Al-Okbi SY, Ammar NM, Mohamed DA (1999). Assessment of the safety of use of certain natural anti-inflammatory agents and their effects on nutritional status in adult and growing rats. *Medical Journal of Islamic Academy of Sciences*.

[30] Latimer KS, Bienzle D, Feldman BF, Zink JG, Jain NC (2000). Determination and interpretation of avian leukogram. *Schalm's Veterinary Hematology*.

[31] Abe M, Akbar F, Hasebe A, Horiike N, Onji M (2003). Glycyrrhizin enhances interleukin-10 production by liver dendritic cells in mice with hepatitis. *Journal of Gastroenterology*.

[32] David L, Hoffman BS

[33] Coles BH, Sutton TB, Swift S (1997). Aids to diagnosis. *Avian Medicine and Surgery*.

[34] Al-Qarawi AA, Abdel-Rahman HA, El-Mougy SA (2001). Hepatoprotective activity of licorice in rat liver injury models. *Journal of Herbs, Spices and Medicinal Plants*.

[35] Guoping L, Cang HH, Li GP, Huang CH (2002). Effects of Chinese medical herbs on controlling piglet early weaning diarrhea. *Chinese Journal of Veterinary Science*.

[36] Ahmed AAS, El Abdin YZ, Hamza S, Saad FE (1975). Effect of experimental duck virus hepatitis infection on some biochemical constituents and enzymes in the serum of white Pekin ducklings. *Avian Diseases*.

[37] Ellakany H, El Sebai AH, Sultan H, Sami AA Control of experimental DHV infection by amantadine.

[38] Kaneko JJ, Harvey JW, Bruss ML (1997). *Clinical Biochemistry of Domestic Animals*.

[39] Nagai T, Egashira T, Kudo Y, Yamanaka Y, Shimada T (1992). Attenuation of dysfunction in the ischemia-reperfused liver by glycyrrhizin. *Japanese Journal of Pharmacology*.

[40] Lee C, Park S, Kim YS (2007). Protective mechanism of glycyrrhizin on acute liver injury induced by carbon tetrachloride in mice. *Biological and Pharmaceutical Bulletin*.

[41] Boll M, Weber LWD, Becker E, Stampfl A (2001). Mechanism of carbon tetrachloride-induced hepatotoxicity. Hepatocellular damage by reactive carbon tetrachloride metabolites. *Zeitschrift fur Naturforschung C*.

[42] Coles EH (1986). *Veterinary Clinical Pathology*.

[43] Murakami T, Uchikawa T (1993). Effect of glycyrrhizine on hyperkalemia due to hyporeninemic hypoaldosteronism in diabetes mellitus. *Life Sciences*.

[44] Haddad PS, Depot M, Settaf A, Cherrah Y (2001). Use of antidiabetic plants in Morocco and Québec. *Diabetes Care*.

[45] Thrall MA, Baker DC, Campbell TW (2004). *Veterinary Hematology and Clinical Chemistry*.

[46] Hochleithner M (1994). *Biochemistries in Avian Medicine (Principles and Application)*.

[47] Shiki Y, Shirai K, Saito Y, Yoshida S, Mori Y, Wakashin M (1992). Effect of glycyrrhizin on lysis of hepatocyte membranes induced by anti-liver cell membrane antibody. *Journal of Gastroenterology and Hepatology*.

